# Letter to the Editor Regarding Kortazar‐Zubizarreta et al. ‘The Risk of Transmission of Genetic Prion Diseases Is Greater Than 50%’

**DOI:** 10.1111/ene.70543

**Published:** 2026-03-02

**Authors:** Akin Nihat, Tze How Mok, Eric Vallabh Minikel, John Collinge, Simon Mead

**Affiliations:** ^1^ MRC Prion Unit at UCL UCL Institute of Prion Diseases London UK; ^2^ NHS National Prion Clinic, National Hospital for Neurology and Neurosurgery University College London Hospitals NHS Foundation Trust London UK; ^3^ Stanley Center for Psychiatric Research Broad Institute Cambridge Massachusetts USA

**Keywords:** Creutzfeldt–Jakob disease, fatal familial insomnia, genetic counselling, prion diseases, transmission rate distortion

Dear Editor,

1

The study by Kortazar‐Zubizarreta et al. [1] presents a provocative challenge to the Mendelian assumption of 50% inheritance of the mutant allele in autosomal dominant prion diseases. Whilst we commend the authors for the first investigation of transmission ratio distortion (TRD) in inherited prion diseases, we have significant concerns regarding the potential for ascertainment bias to have inflated the reported transmission rates of 67%–70%. We urge caution regarding these conclusions and do not support integrating findings into patient information or genetic counselling protocols.

The study recruited from specialised neurology or genetic counselling services at a single site and the Spanish Prion Disease Foundation. This ascertainment approach selects for a series of events more likely to occur in families with greater disease burden or demonstrated transmission: contact with specialist services, recognition of a familial prion disease pedigree and uptake of genetic testing in offspring. Even if some non‐transmitting families are identified, the probability of inclusion is clearly differential with respect to transmission. This concern could be mitigated if every member of a nuclear family was exhaustively genotyped or if inclusion were restricted to those undertaking predictive testing; however, neither of these approaches was employed. In their absence, the opportunistic inclusion of individuals whose genotype was discovered only because they presented with disease is likely to select for offspring in which transmission has already occurred.

Furthermore, the inclusion of ‘obligate carriers’ creates a circular logic likely to inflate transmission estimates. By identifying patients only after transmission has already occurred in offspring, or via post‐mortem genotyping (more likely to be undertaken after another family member is affected), these methods capture instances of transmission while remaining blind to non‐transmission.

The exclusion criteria are also likely to have systematically omitted families with low or no transmission. Excluded families consisting entirely of unaffected individuals where a parent carrier could not be identified and families where offspring did not undergo genotyping comprised nearly 30% of the originally eligible cohort. Considering the study sample size and wide confidence intervals around estimated transmission rates, adding even a few nuclear families without transmission would likely reduce group‐level estimates to within the expected margin of statistical error of Mendelian transmission, particularly in sex‐stratified analyses.

The authors argue that the effect of ascertainment bias is negligible, citing that 35.4% of included nuclear families had transmission rates at or below 50%. However, this does not equate to unbiased ascertainment—selection processes can readily inflate the mean transmission estimate even while leaving a substantial proportion of families below 50%, particularly when inclusion is triggered by the presence of an affected or genotyped carrier within a lineage.

Learning that they are at risk of inherited prion disease is a devastating experience for families. Patients rely on clinicians to provide reliable information regarding transmission risk based on robust, unbiased data. We are concerned that the much higher risk estimates presented here, derived from a cohort that demonstrably omits cases of non‐transmission, may cause undue alarm and confusion for families navigating the considerable challenges already posed by these conditions. Unless these findings are replicated in large, prospective, population‐based cohorts with more stringent case ascertainment criteria, we argue that the 50% Mendelian risk should remain the ethically and statistically sound standard for genetic counselling [2].

## Author Contributions

**Akin Nihat:** conceptualization, writing – original draft, writing – review and editing. **Eric Vallabh Minikel:** conceptualization, writing – review and editing.

## Funding

This work was supported by the Medical Research Council.

## Conflicts of Interest

J.C. is a Director of D‐Gen Ltd., an academic spin‐out company working in the field of prion disease diagnosis, decontamination and therapeutics. E.V.M. has received speaking fees from Abbvie, Eli Lilly, Novartis, Vertex and Voyager; consulting fees from Alnylam, Deerfield and Regeneron; and research support from Cenos, Eli Lilly, Gate Bio, Ionis and Sangamo Therapeutics.

## Data Availability

The authors have nothing to report.
